# Can we rely on out-of-hospital blood samples? A prospective interventional study on the pre-analytical stability of blood samples under prehospital emergency medicine conditions

**DOI:** 10.1186/s13049-017-0371-3

**Published:** 2017-03-04

**Authors:** Johannes Prottengeier, Nicola Jess, Frank Harig, Christine Gall, Joachim Schmidt, Torsten Birkholz

**Affiliations:** 1 Department of Anaesthesiology, Erlangen University Hospital, Krankenhausstrasse 12, 91054 Erlangen, Germany; 2 Department of Child and Adolescent Psychiatry, Psychosomatics, and Psychotherapy, Ludwig-Maximilians-University, Munich, Germany; 3 Department of Cardiac Surgery, Erlangen University Hospital, Erlangen, Germany; 4 Department of Medical Informatics, Biometry and Epidemiology, Friedrich Alexander University, Erlangen-Nuremberg, Erlangen, Germany

**Keywords:** Blood sampling, Sample handling, Preanalytical stability, Prehospital emergency medicine

## Abstract

**Background:**

Prehospital intravenous access provides the opportunity to sample blood from an emergency patient at the earliest possible moment in the course of acute illness and in a state prior to therapeutic interventions. Our study investigates the pre-analytical stability of biomarkers in prehospital emergency medicine and will answer the question whether an approach of blood sampling out in the field will deliver valid laboratory results.

**Methods:**

We prepared pairs of blood samples from healthy volunteers and volunteering patients post cardio-thoracic surgery. While one sample set was analysed immediately, the other one was subjected to a worse-than-reality treatment of 60 min time-lapse and standardized mechanical forces outside of the hospital through actual ambulance transport. We investigated 21 parameters comprising blood cells, coagulation tests, electrolytes, markers of haemolysis and markers of cardiac ischemia. Bland-Altman analysis was used to investigate differences between test groups. Differences between test groups were set against the official margins of test accuracy as given by the German Requirements for Quality Assurance of Medical Laboratory Examinations.

**Results:**

Agreement between immediate analysis and our prehospital treatment is high as demonstrated by Bland-Altman plotting. Mechanical stress and time delay do not produce a systematic bias but only random inaccuracy. The limits of agreement for the tested parameters are generally within clinically acceptable ranges of variation and within the official margins as set by the German Requirements for Quality Assurance of Medical Laboratory Examinations.

**Discussion:**

We subjected blood samples to a standardized treatment marking a worse-than-reality scenario of prehospital time delay and transport. Biomarkers including indicators of myocardial ischemia showed high pre-analytical stability.

**Conclusion:**

We conclude the validity of blood samples from a prehospital environment.

**Electronic supplementary material:**

The online version of this article (doi:10.1186/s13049-017-0371-3) contains supplementary material, which is available to authorized users.

## Background

Laboratory tests from blood samples are a major component in the diagnostic work-up of emergency patients [1, 2]. Usually the required blood samples are drawn inside the emergency department (ED) and there is a general consensus, that samples should be analysed as soon as possible and handled carefully [3–5]. Whenever a patient is being cared for by emergency medical services (EMS) prior to his admission to the ED, there arises an opportunity and unsolved question at the same time: EMS will frequently establish venous access [6, 7]. This could very well be utilized to secure blood specimens back-to-back to a patient’s first contact with medical help. Firstly, such samples would produce results representative of the patient’s condition before any intervention: before infusion of fluids and haemodilution, before heparin and anticoagulation, before diuretics and changes to electrolytes – to mention a few iatrogenic alterations. Secondly they would provide additional insight into the course of dynamic biomarkers in scenarios such as myocardial infarction [8]. And finally, prehospital sampling may save time in the diagnostic process as time consumption for blood sampling adjacent to the process of venous cannulation may be smaller than a second procedure performed inside the ED, especially considering the inversion of the patient-staff ratio from the prehospital to the intrahospital setting.

But are those presumed advantages real? Up to date no data exists to evaluate the clinical value of prehospital blood sampling. Even less, literature provides no answer to the more basic question, whether those laboratory values relevant to emergency medicine show the necessary preanalytical stability to allow for reliable testing–if they were gathered in a prehospital setting.

The aim of our study was to subject blood samples to a standardized “worst-case-scenario” prehospital treatment of mechanical forces and time delay before laboratory testing. We investigated the preanalytical stability of biomarkers frequently used in emergency diagnostics compared to an immediate analysis. This included myocardial enzymes, complete blood counts and tests of haemostasis, haemolysis, electrolytes and different proteins. Owing to its key role in the work-up of “chest-pain” we focused on the changes of troponin levels as our primary parameter of interest.

## Methods

### Blood donors

This study was approved by the ethics board of the University of Erlangen-Nuremberg. Blood sampling took place at the Erlangen University Hospital. Samples where provided by healthy volunteers (physicians, nurses, paramedics) and volunteering scheduled patients post major cardio-thoracic surgery. All volunteers gave their informed consent well before the investigations. The composition of our study population guaranteed that biomarkers covered a wide range of values from physiological (in healthy volunteers) to highly pathological (e.g. elevated cardiac enzymes in patients post cardiotomy).

### Blood sampling

All samples were collected under standardized, stringently timed conditions and adherence to the syringe manufacturers’ product manuals and the hospital’s standard operating procedures of hygiene and safety. Only venous blood was obtained. Specimens for blood gas analysis were contained in “safePico Aspirators” (Radiometer Medical ApS, Brønshøj, Denmark) and all other specimen in “S-Monovettes” (Sarstedt AG, Nümbrecht, Germany). Table 1 shows the allocation of parameters to their specific syringe. Analysis took place in the University Hospital’s laboratories with the exception of blood gas analyses, which were performed on the Department of Anaesthesia’s own ABL 800Flex analysers (Radiometer Medical ApS, Brønshøj, Denmark). Table 2 provides an overview of analysers, analytical methods and legally required analytical accuracy for each parameter.

**Table 1:** List of parameters and sample syringes

Type of syringe	S-Monovette/red Haematology	S-Monovette/green Coagulation profile	S-Monovette/white Clotting Activator/Serum	S-Monovette/orange Lithium Heparin/Serum	safePICO Aspirator Blood-Gas-Analysis
Additive	Potassium-EDTA	Citrate	Clotting Activator	Lithium Heparin	80 IU Heparin
Parameters (alphabetic order) of laboratory testing	Haemoglobin	Antithrombin III	*Alpha-HBDH*	*Alpha-HBDH*	Calcium/Ca++
	Leukocytes	Activated Partial Thromboplastin Time (aPTT)	Creatinine	Creatinine	Chloride/Cl-
	Thrombocytes	Fibrinogen	Creatin Kinase/CK	Creatin Kinase/CK	Potassium/K^+^
		International Normalized Ratio (INR)	*Free Haemoglobin*	*Free Haemoglobin*	Sodium/Na^+^
			Glutamic Oxaloacetic Transaminase	Glutamic Oxaloacetic Transaminase	
				Troponin I

**Table 2:** Biomarkers and their analysers, analytical methods and quality criteria

Biomarkers in alphabetic order	Analyser model	Manufacturer	Test method	Frequency of quality controls (3 shifts per day.)	Acceptable Root Mean Standard Deviation as defined by Rili-BAEK	Range of interest as defined by Rili-BAEK	Unit
Alpha-HBDH	AU 680	Beckman Coulter Inc., Brea, United States	Kinetic (decreasing) UV-Test at 37 °C	Twice per 8 h-shift plus after every 50 specimen.	not defined by Rili-BAEK	not defined by Rili-BAEK	U/l
Antithrombin III	STA-R Evolution	Diagnostica Stago S.A.S., Asnieres sur Seine, France	Colorimetric Assay (ELISA) of Antithrombin III Activity	4 times per 8 h-shift.	not defined by Rili-BAEK	not defined by Rili-BAEK	U/l
aPTT	STA-R Evolution	Diagnostica Stago S.A.S., Asnieres sur Seine, France	Time to clotting after recalcification of plasma in the presence of cephalin (platelet substitute) and a factor XII activator (poly-phenolic component)	4 times per 8 h-shift.	10,5%	20–120	sec.
Calcium (ionized)	ABL 800Flex	Radiometer Medical ApS, Brønshøj, Denmark	Potentiometric method	Once per 8 h-shift.	14%	0,2–≤ 1	mmol/l
					7,5%	>1–2,5	
Chloride	ABL 800Flex	Radiometer Medical ApS, Brønshøj, Denmark	Potentiometric method	Once per 8 h-shift.	4,5%	70–150	mmol/l
Creatin Kinase (CK)	AU 680	Beckman Coulter Inc., Brea, United States	International Federation of Clinical Chemistry and Laboratory Medicine Method	Twice per 8 h-shift plus after every 50 specimen.	11%	50–1000	U/l
Creatinine	AU 680	Beckman Coulter Inc., Brea, United States	Kinetic colorimetric Jaffe reaction	Twice per 8 h-shift plus after every 50 specimen.	11,5%	0,5–10	mg/dl
Fibrinogen	STA-R Evolution	Diagnostica Stago S.A.S., Asnieres sur Seine, France	Clauss clotting assay	4 times per 8 h-shift.	not defined by Rili-BAEK	not defined by Rili-BAEK	mg/dl
Free Haemoglobin	Cobas Integra 400	Hoffmann- La Roche AG, Basel, Switzerland	Colorimetric Test	4 times per 8 h-shift.	20%	not defined by Rili-BAEK	mg/l
Glutamic Oxaloacetic Transaminase (GOT/AST)	AU 680	Beckman Coulter Inc., Brea, United States	International Federation of Clinical Chemistry and Laboratory Medicine - Method	Twice per 8 h-shift plus after every 50 specimen.	11,5%	20–400	U/l
Haemoglobin	XE5000	Sysmex Corporation KK, Kobe, Japan	SLS (sodium lauryl sulphate) haemoglobin method	Once per 8 h-shift.	4%	2–20	g/dl
INR	STA-R Evolution	Diagnostica Stago S.A.S., Asnieres sur Seine, France	Time to clotting after addition of Calcium-rich thromboplastine (Neoplastine CI plus)	4 times per 8 h-shift.	not defined by Rili-BAEK	not defined by Rili-BAEK	n.n.
Leukocytes	XE5000	Sysmex Corporation KK, Kobe, Japan	Flow cytometry with dynamic focusing	Once per 8 h-shift.	6,5%	2–30	10^9^/l
Myoglobin	AU 5800	Beckman Coulter Inc., Brea, United States	electrochemiluminescence-immunoassay	Twice per 8-h shift	not defined by Rili-BAEK	not defined by Rili-BAEK	μg/l
Potassium	ABL 800Flex	Radiometer Medical ApS, Brønshøj, Denmark	Potentiometric method	Once per 8 h-shift.	4,5%	2–8	mmol/l
Sodium	ABL 800Flex	Radiometer Medical ApS, Brønshøj, Denmark	Potentiometric method	Once per 8 h-shift.	3%	110–180	mmol/l
Thrombocytes	XE5000	Sysmex Corporation KK, Kobe, Japan	Flow cytometry with dynamic focusing; traditional impedance technology for very low and very high PLT counts	Once per 8 h-shift.	13,5%	40–≤150	10^9^/l
					8,5%	150–≤300	
					7,5%	300–700	
Troponin I	Access 2	Beckman Coulter Inc., Brea, United States	electrochemiluminescence immunoassay	4 times per 8 h-shift.	20%	0,1–35	μg/l

For more detailed information on the laboratory testing of our selected parameters we refer to the technical manuals and reference guidebooks of each analyser as well as the Guideline of the German Medical Association on Quality Assurance in Medical Laboratory Examinations (Rili-BAEK [15])

We generated two equal sets of syringes from every volunteer. Parameters can be divided in two groups: Group 1 represents a shortlist of biomarkers highly relevant to emergency medicine. Group 2 (items *in italics* in Table 1) represent markers of haemolysis which may falsify other test procedures.

Each set of samples was allotted to one of two different treatment conditions.

### Control Group (CG)

All specimen were immediately transported to analysis by foot within 10 min of sampling. All specimens were subject to room temperature as provided by the hospital’s climate control system.

### Prehospital Group (PHG)

Samples were analysed after a time lapse of 60 min during which they underwent a standardized scenario of mechanical stress (see below). During transport all samples were kept lying flat in a cooler box (Coleman Inc., Wichita, United States) tempered to the intensive care unit’s 23 °C standard room temperature, which is maintained by a climate control system. Temperature of the test compartment was monitored end-to-end and samples were not subject to external heating or cooling. By these means of storage, samples were also shielded from solar radiation.

### Prehospital scenario

Samples allotted to the PHG were transferred to an ambulance truck (1998 Sprinter, Model 312 D, Mercedes Benz, Stuttgart, Germany. It was the oldest and most worn down model available from local emergency services at that time.). The Cooler Box was placed on the worktop inside the patient’s cabin. Samples were sent onto a 15 km and 30 min long drive in and around the city of Erlangen, which consisted of city traffic, dirt tracks and larger state roads. Driving mode (gear), velocity, and thus time consumption and the quantity of mechanical forces were standardized through a dedicated driving protocol. The reproducibility of mechanical forces was evaluated in pre-test trials using a Triaxial Vibration Meter VM30-H (Metra Mess- und Frequenztechnik, Radebeul, Germany) placed in the site of the syringes.

Under the infrastructural and geographic circumstances of German EMS, pre-hospital time requirements shall not exceed 60 min. This time frame has originated from the “golden hour of shock” and has been adopted for time-sensitive diagnoses such as multiple trauma, traumatic brain injury, stroke, sepsis, chest-pain and resuscitation. It is the upper limit for the pre-hospital phase of emergency care in Germany and is one of the main determinants for the allocation of EMS resources [9].

In context of these circumstances, our prehospital set-up represented a worst-case scenario by far longer in duration and far more mechanically straining than average missions found in land-based EMS in Germany [10–13].

### Data analysis and statistics

To analyse the effect of mechanical stress and time on blood samples, we use Bland-Altman plots to compare the measurements with the reference blood samples which were analysed immediately [14].

The solid line in the graph shows the mean of the differences. The dashed upper (lower) line shows the upper (lower) limit of agreement equal to mean ± 2SD. Within these limits, typically lie 95% of the samples differences. The dotted lines represent the respective 95% confidence intervals. As external margins for negligible differences, we use the relative differences specified for certain ranges of validity given by the Guidelines of the German Medical Association on quality assurance in medical laboratory testing (Rili-BAEK) [15]. The Rili-BAEK is the official benchmarking guideline of German medical laboratories. The validity of test results is strictly limited by predefined margins of error. Therefore we postulate that results must be considered as equal, should their differences lie within the set margins of the Rili-BAEK.

Comparing Bland-Altman plots for absolute and relative differences confirmed our choice of absolute differences as then the points better fit the horizontal limits of agreement along the range of the x-axis. Thus, the tolerated difference given by Rili-BAEK [15] is not constant over x but shown by sloping lines.

Analyses were performed in R 3.2.4 [R Core Team (2013). R: A language and environment for statistical computing. R Foundation for Statistical Computing, Vienna, Austria. URL http://www.R-project.org/].

## Results

We prepared 134 sample sets and selected 21 biomarkers for Bland-Altman analysis (Table 3). Factors such as analyser malfunction, treatment against protocol (dropping of syringes, inadequate filling heights etc.) led to the exclusion of some samples. The number of complete data sets for each parameter is documented.

**Table 3:** Overview of analysed biomarkers

Biomarkers in alphabetic order	Unit	Number of measurements	Mean of differences	CI mean	Upper limit of agreement	CI upper limit of agreement	Lower limit of agreement	CI lower limit of agreement
Alpha-HBDH (Clotting Activator)	U/l	133	−1,143	[−6.38; 4.095]	59,928	[50.857;69]	−62,214	[−71.286; −53.142]
Alpha-HBDH (Lithium Heparin)	U/l	133	6,474	[2.725; 10.223]	50,189	[43.695;56.682]	−37,241	[−43.735; −30.748]
Antithrombin III	U/l	128	−0,156	[−0.858; 0.546]	7,869	[6.653;9.085]	−8,182	[−9.397; −6.966]
aPTT	sec.	128	−0,049	[−0.318; 0.22]	3,029	[2.563;3.495]	−3,128	[−3.594; −2.661]
Calcium	mmol/l	129	0,004	[−0.001; 0.009]	0,063	[0.054;0.072]	−0,055	[−0.064; −0.046]
Chloride	mmol/l	131	−0,191	[−0.424; 0.042]	2,505	[2.102;2.909]	−2,887	[−3.29; −2.483]
Creatin Kinase (CK) (Clotting Activator)	U/l	133	0,15	[−0.977; 1.278]	13,301	[11.348;15.254]	−13	[−14.954; −11.047]
Creatin Kinase (CK) (Lithium Heparin)	U/l	133	−0,895	[−2.044; 0.255]	12,509	[10.518;14.5]	−14,298	[−16.289; −12.307]
Creatinine	mg/dl	134	−0,006	[−0.01; −0.001]	0,046	[0.039;0.054]	−0,058	[−0.066; −0.05]
Fibrinogen	mg/dl	128	2,438	[−3.973; 8.848]	75,743	[64.639;86.847]	−70,868	[−81.972; −59.764]
Free Haemoglobin (Clotting Activator)	mg/l	132	−0,606	[−2.528; 1.316]	21,718	[18.389;25.047]	−22,93	[−26.259; −19.601]
Free Haemoglobin (Lithium Heparin)	mg/l	128	−0,062	[−1.729; 1.604]	18,992	[16.106;21.878]	−19,117	[−22.003; −16.231]
Glutamic Oxaloacetic Transaminase (GOT/AST)	U/l	134	0,604	[0.207; 1.001]	5,252	[4.564;5.939]	−4,043	[−4.73; −3.355]
Haemoglobin	g/dl	131	0,005	[−0.033; 0.044]	0,45	[0.383;0.517]	−0,439	[−0.506; −0.373]
INR		128	−0,003	[−0.009; 0.002]	0,059	[0.05;0.069]	−0,066	[−0.075; −0.056]
Leukocytes	10^9^/l	131	−0,005	[−0.067; 0.057]	0,713	[0.605;0.82]	−0,722	[−0.829; −0.615]
Myoglobin	μg/l	134	−2,336	[−4.019; −0.652]	17,367	[14.452;20.283]	−22,039	[−24.954; −19.123]
Potassium	mmol/l	129	0,017	[−0.013; 0.046]	0,355	[0.304;0.406]	−0,321	[−0.372; −0.27]
Sodium	mmol/l	129	0,227	[−0.011; 0.465]	2,956	[2.544;3.368]	−2,502	[−2.914; −2.09]
Thrombocytes	10^9^/l	131	6,443	[4.765; 8.12]	25,85	[22.944;28.755]	−12,964	[−15.869; −10.059]
Troponin I (Lithium Heparin)	μg/l	134	−0,009	[−0.016; −0.002]	0,072	[0.06;0.084]	−0,091	[−0.103; −0.079]

Means of differences, standard deviations and confidence intervals between Groups of immediate analysis and analysis after mechanical stress and time delay. The estimated means of differences are always negligible with a small 95%-CI, i.e. mechanical stress does not produce a systematic bias but only random inaccuracy

**Fig. 1:**
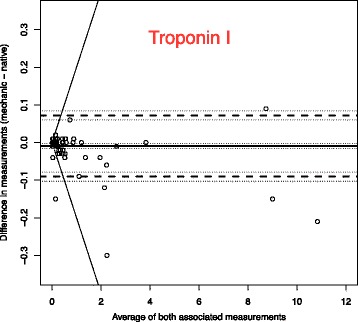
Troponin I (Unit: ng/dl)–One outlying sample pair has been excluded from the plot for reasons of better graphical display (Native: 36.82; Mechanic: 37.22). Bland-Altman plots for selected biomarkers. The difference in measurements is plotted against the average value of both associated measurements. The margins of accuracy as given by the Rili-BAEK are drawn as sloping lines. Agreement is high. Variations are random and no systemical bias through treatment can be detected. Inaccuracy is well within the limits of the Rili-BAEK and within tolerance of clinical interpretation

**Fig. 2:**
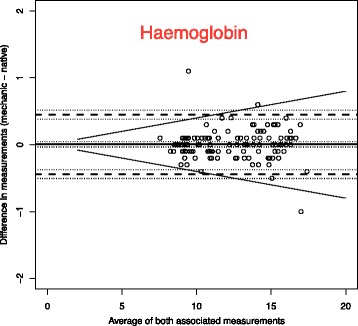
Haemoglobin (Unit: g/dl). Bland-Altman plots for selected biomarkers. The difference in measurements is plotted against the average value of both associated measurements. The margins of accuracy as given by the Rili-BAEK are drawn as sloping lines. Agreement is high. Variations are random and no systemical bias through treatment can be detected. Inaccuracy is well within the limits of the Rili-BAEK and within tolerance of clinical interpretation

**Fig. 3:**
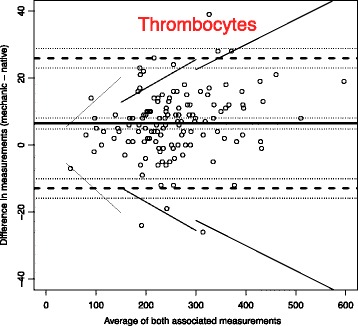
Thrombocytes (Unit: 10^9^/l). Bland-Altman plots for selected biomarkers. The difference in measurements is plotted against the average value of both associated measurements. The margins of accuracy as given by the Rili-BAEK are drawn as sloping lines. Agreement is high. Variations are random and no systemical bias through treatment can be detected. Inaccuracy is well within the limits of the Rili-BAEK and within tolerance of clinical interpretation

**Fig. 4:**
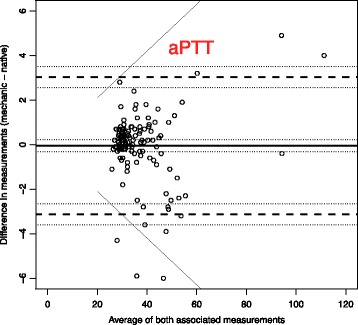
Activated Partial Thromboplastin Time – aPTT (Unit: sec.). Bland-Altman plots for selected biomarkers. The difference in measurements is plotted against the average value of both associated measurements. The margins of accuracy as given by the Rili-BAEK are drawn as sloping lines. Agreement is high. Variations are random and no systemical bias through treatment can be detected. Inaccuracy is well within the limits of the Rili-BAEK and within tolerance of clinical interpretation

**Fig. 5:**
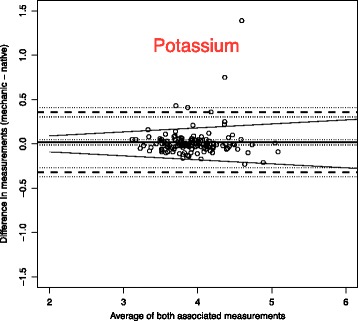
Potassium (Unit: mmol/l). Bland-Altman plots for selected biomarkers. The difference in measurements is plotted against the average value of both associated measurements. The margins of accuracy as given by the Rili-BAEK are drawn as sloping lines. Agreement is high. Variations are random and no systemical bias through treatment can be detected. Inaccuracy is well within the limits of the Rili-BAEK and within tolerance of clinical interpretation

**Fig. 6:**
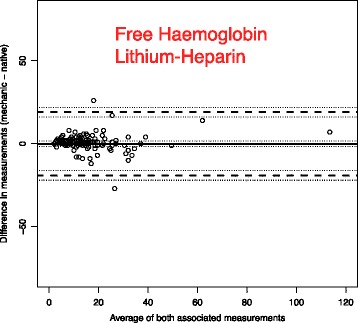
Free Haemoglobin in Lithium-Heparin preserved Serum (Unit: mg/l). Bland-Altman plots for selected biomarkers. The difference in measurements is plotted against the average value of both associated measurements. The margins of accuracy as given by the Rili-BAEK are drawn as sloping lines. Agreement is high. Variations are random and no systemical bias through treatment can be detected. Inaccuracy is well within the limits of the Rili-BAEK and within tolerance of clinical interpretation

The effect of our prehospital scenario compared to immediate testing is demonstrated by the Bland-Altman plots of each parameter. We present in this manuscript a selection of plots in Figs. 1, 2, 3, 4, 5 and 6. Plots for 8 other analysed parameters can be found as Additional files 1, 2, 3, 4, 5, 6, 7 and 8 (Figures S7–S14). Table 3 provides a comprehensive overview of each parameter, means of differences, standard deviations and confidence intervals between Groups CG and PHG. The estimated means of differences are always negligible with a small 95%-CI, i.e. mechanical stress does not produce a systematic bias but only random inaccuracy. The limits of agreement are universally within clinically acceptable ranges of variation. Alterations are mostly within the official margin of accuracy as set by the Rili-BAEK. Troponin I showed comparable results when compared between immediate analysis and analysis after the combination of time delay and mechanical stress. Among the 134 samples, 47/134 = 35% of the troponin values lie within the interval from 0.1 to 35 ng/dl which is the range addressed by the Rili-BAEK. Only for two samples, the relative difference was more than 0.2 ng/dl, precisely 0.3 and 0.21 ng/dl. For 15 (=15/47 = 32%) of these 47 values, there was no measurable difference at all, i.e. the measurement after prehospital treatment was exactly like the immediately analysed one. For the rest of the values, the relative difference was less or equal to 0.15 ng/dl. Thus, for 45/47 = 95,7% (CI [89.97%; 100%]) of the samples, the relative difference is easily acceptable. For 87/134 = 65% of the samples, the troponin measurements are lower than 0.1 ng/dl. For 78 (=89.655%, CI = [83.256%; 96.1%]) samples, the deviation is Zero or less than the technical capabilities of measurement, respectively. For seven samples, the absolute differences are equal to 0.01 ng/dl. For the remaining two samples, they are 0.04 and 0.4 ng/dl.

Haemolysis as a potent interference factor with many chromage laboratory tests was investigated and excluded in relevant levels by investigations into alpha-HBDH and free haemoglobin in serum (Fig. 6 and Additional files 6, 7 and 8: Figures S12–S14).

## Discussion

Emergency services regularly represent the first line of contact between medical care and patients. In many cases, circumstances would allow for the sampling of blood in the early stages of acute illness. While it is conceivable that such an approach might have clinical benefits, there is little data to proof the pre-analytical validity of biomarkers achieved this way. In our study we simulated the prehospital fate of blood samples and investigated changes to test results when compared to immediate analysis. We improvised a combination of time delay and mechanical stress that resulted in a sample exposition worse than what would be expected in German EMS on a regular basis.

We found that our exposition of specimens towards time delay and mechanical stress caused only small changes to the parameters, as visualised in the Bland-Altman-Plots. However, the seemingly simple question whether the plotted differences may be considered as “same or different” cannot be answered by a universally accepted formula. The interpretation of bare values actually depends on the overall clinical context and many factors more.

The definition of “analytical validity” of the laboratory test itself also deals with this challenge and provides guidance for our dilemma. Definitions and margins derive from technical aspects. They are generally accepted and uncoupled from subjective interpretation. Even more so, they are given at a level of precision that guarantees a reliable foundation for the diagnostic process in general. Stringent quality control is mandatory and defines clear margins for the accuracy of analytical testing for each biomarker (Rili-BAEK) [15]. As a consequence it has to be postulated, that test results with differences between them that lie within these limits of test accuracy must be accepted as equal – technically and clinically.

Within our data set the differences between treatment groups generally lie within those margins of test accuracy. Therefore results after prehospital treatment can be considered as clinically equal to those from immediate analysis. The pre-analytical validity is given under conditions of a worst-case scenario and therefore we postulate even smaller inaccuracies under average mission circumstances in Germany. Stability is given in all types of syringes. It is demonstrated for cell counts, coagulation profiles, electrolyte levels and protein serum biomarkers. Our assortment of tests represents some of the most relevant biomarkers in emergency medicine and covers a wide range of disorders.

Haemolysis may interfere with chromagen tests, but has been ruled out as a confounder by stable results for alpha-HBDH and free haemoglobin during our experiments [16, 17].

These findings are in accordance with previous studies of pre-analytical stability of biomarkers in other surroundings. Troponin shows preanalytical validity in a variety of maltreatment scenarios such as prolonged or sub-par storage and repeated freeze-thaw cycles [18–20]. However, troponin is a protein of limited metabolism within the confinements of a blood sample and results may vary with biomarkers of different biological properties.

Rotational thromboelastometry for example represents a multifactorial functional test that is highly susceptible to mechanical interference during analysis, and has been shown to be subject to pre-analytical influences as well. Transport of samples through pneumatic tube systems led to statistically significant alterations of test results. However, those changes were all within limits of what the investigators defined as clinical irrelevance [21, 22].

Furthermore, some biomarkers need to be excluded from analysis owing to biophysical considerations. Volatile solubles such as oxygen and carbon dioxide will rapidly alter their concentrations with clinical relevance only minutes after sampling [23, 24].

In the end the clinical relevance of our study derives from three points. 1. Our analysis brings alterations into the accepted context of analytical accuracy as outlined by the quality-assurance guidelines of the “Rili-BAEK”. 2. Treatment of samples represents a worst-case scenario and suggests less error with normal treatment. 3. Our choice of parameters reflects on relevance to emergency medicine and excludes those *a priori* unsuitable due to biophysical reasons.

Naturally there are some limitations to our study. As a first, our specific scenario to test sample integrity represents only one combination of stressors out of many thinkable. Our main focus lay on time delay and mechanical forces as possible factors of interference. However, other external circumstances may influence preanalytical stability as well such as subpar conditions for blood sampling itself, extreme variations in temperature and exposure to solar radiation. As pre-hospital emergency medicine regularly takes place in an improvised outdoor environment, these influences should be considered and investigated in future studies. However, our study also demonstrates, that low-tech solutions such as the use of a cooler-box for sample storage can substantially limit the impact of environmental elements.

Secondly, we have to admit that specimen treatment might differ in nuances from ride to ride – in spite of all steps taken to provide for reproducibility. However, these limitations seem agreeable when compared to the alternative of–highly standardizable, but totally artificial–mechanical stress generated by vibrating devices inside a laboratory.

Thirdly, our exposition scenario is aligned towards transportation times found in the well-developed EMS-infrastructure of a densely-populated country. It does not cover very long sample transportation times beyond 60 min as they might be found in geographically challenging settings or under conditions of limited health-care infrastructure [25].

As a forth, we can only draw conclusions from parameters within the range of our test samples. While it is biologically unlikely that extremely high or low concentrations of biomarkers will have different reactions to our scenario, the absence of extreme values in our data set does not allow anything but speculation for such outliers.

Finally, our investigation focused on a selection of parameters and methods of analysis and we can only provide data for those within this collection.

## Conclusion

Prehospital emergency medicine offers the possibility to sample blood in the early stages of acute illness. Our study demonstrates the pre-analytical stability and validity of samples of such origin even after time delay and transport outside of the hospital. The data set includes markers of myocardial ischemia such as troponin and creatine kinase, next to blood cells, electrolytes and coagulation profiles. Keeping in mind the limitations of this investigation, it seems worthwhile to investigate through future studies, the possible benefits to patient outcome deriving from out-of-hospital blood sampling.

## Additional files

Additional file 1: Figure S1. Leukocytes (Unit: 10^9^/l). Bland-Altman plots for selected biomarkers. The difference in measurements is plotted against the average value of both associated measurements. The margins of accuracy as given by the Rili-BAEK are drawn as sloping lines. Agreement is high. Variations are random and no systemical bias through treatment can be detected. Inaccuracy is well within the limits of the Rili-BAEK and within tolerance of clinical interpretation. (PDF 37 kb)
Additional file 2: Figure S2. INR (Unit: not applicable). Bland-Altman plots for selected biomarkers. The difference in measurements is plotted against the average value of both associated measurements. The margins of accuracy as given by the Rili-BAEK are drawn as sloping lines. Agreement is high. Variations are random and no systemical bias through treatment can be detected. Inaccuracy is well within the limits of the Rili-BAEK and within tolerance of clinical interpretation. (PDF 36 kb)
Additional file 3: Figure S3. Creatin Kinase in serum with clotting activator (Unit: U/l). Bland-Altman plots for selected biomarkers. The difference in measurements is plotted against the average value of both associated measurements. The margins of accuracy as given by the Rili-BAEK are drawn as sloping lines. Agreement is high. Variations are random and no systemical bias through treatment can be detected. Inaccuracy is well within the limits of the Rili-BAEK and within tolerance of clinical interpretation. (PDF 63 kb)
Additional file 4: Figure S4. Creatin Kinase in Lithium-Heparin preserved serum (Unit: U/l). Bland-Altman plots for selected biomarkers. The difference in measurements is plotted against the average value of both associated measurements. The margins of accuracy as given by the Rili-BAEK are drawn as sloping lines. Agreement is high. Variations are random and no systemical bias through treatment can be detected. Inaccuracy is well within the limits of the Rili-BAEK and within tolerance of clinical interpretation. (PDF 94 kb)
Additional file 5: Figure S5. Creatinine (Unit: mg/dl). Bland-Altman plots for selected biomarkers. The difference in measurements is plotted against the average value of both associated measurements. The margins of accuracy as given by the Rili-BAEK are drawn as sloping lines. Agreement is high. Variations are random and no systemical bias through treatment can be detected. Inaccuracy is well within the limits of the Rili-BAEK and within tolerance of clinical interpretation. (PDF 154 kb)
Additional file 6: Figure S6. Free Haemoglobin in serum with clotting activator (Unit: mg/l). Bland-Altman plots for selected biomarkers. The difference in measurements is plotted against the average value of both associated measurements. The margins of accuracy as given by the Rili-BAEK are drawn as sloping lines. Agreement is high. Variations are random and no systemical bias through treatment can be detected. Inaccuracy is well within the limits of the Rili-BAEK and within tolerance of clinical interpretation. (PDF 286 kb)
Additional file 7: Figure S7. Alpha-HBDH in serum with clotting activator (Unit: U/l) - Two outlying sample pairs have been excluded from the plot for reasons of better graphical display (No. 1: Native: 1532; Mechanic: 1529; No. 2: Native 357: Mechanic: 135). Bland-Altman plots for selected biomarkers. The difference in measurements is plotted against the average value of both associated measurements. The margins of accuracy as given by the Rili-BAEK are drawn as sloping lines. Agreement is high. Variations are random and no systemical bias through treatment can be detected. Inaccuracy is well within the limits of the Rili-BAEK and within tolerance of clinical interpretation. (PDF 566 kb)
Additional file 8: Figure S8. Alpha-HBDH in Lithium-Heparin preserved serum (Unit: U/l)–One outlying sample pair has been excluded from the plot for reasons of better graphical display (Native: 1526; Mechanic: 1534). Bland-Altman plots for selected biomarkers. The difference in measurements is plotted against the average value of both associated measurements. The margins of accuracy as given by the Rili-BAEK are drawn as sloping lines. Agreement is high. Variations are random and no systemical bias through treatment can be detected. Inaccuracy is well within the limits of the Rili-BAEK and within tolerance of clinical interpretation. (PDF 38 kb)

## Abbreviations

CG: Control Group
ED: Emergency department
EMS: Emergency medical service
PHG: Pre-Hospital-Group
Rili-BAEK: Guidelines of the German Medical Association on quality assurance in medical laboratory testing
