# Supplementary figures and images for: Can we rely on out-of-hospital blood samples? A prospective interventional study on the pre-analytical stability of blood samples under prehospital emergency medicine conditions
Source: Scand J Trauma Resusc Emerg Med. 2017 Mar 4;25:24. doi: 10.1186/s13049-017-0371-3 (PMC5336613; doi:10.1186/s13049-017-0371-3)

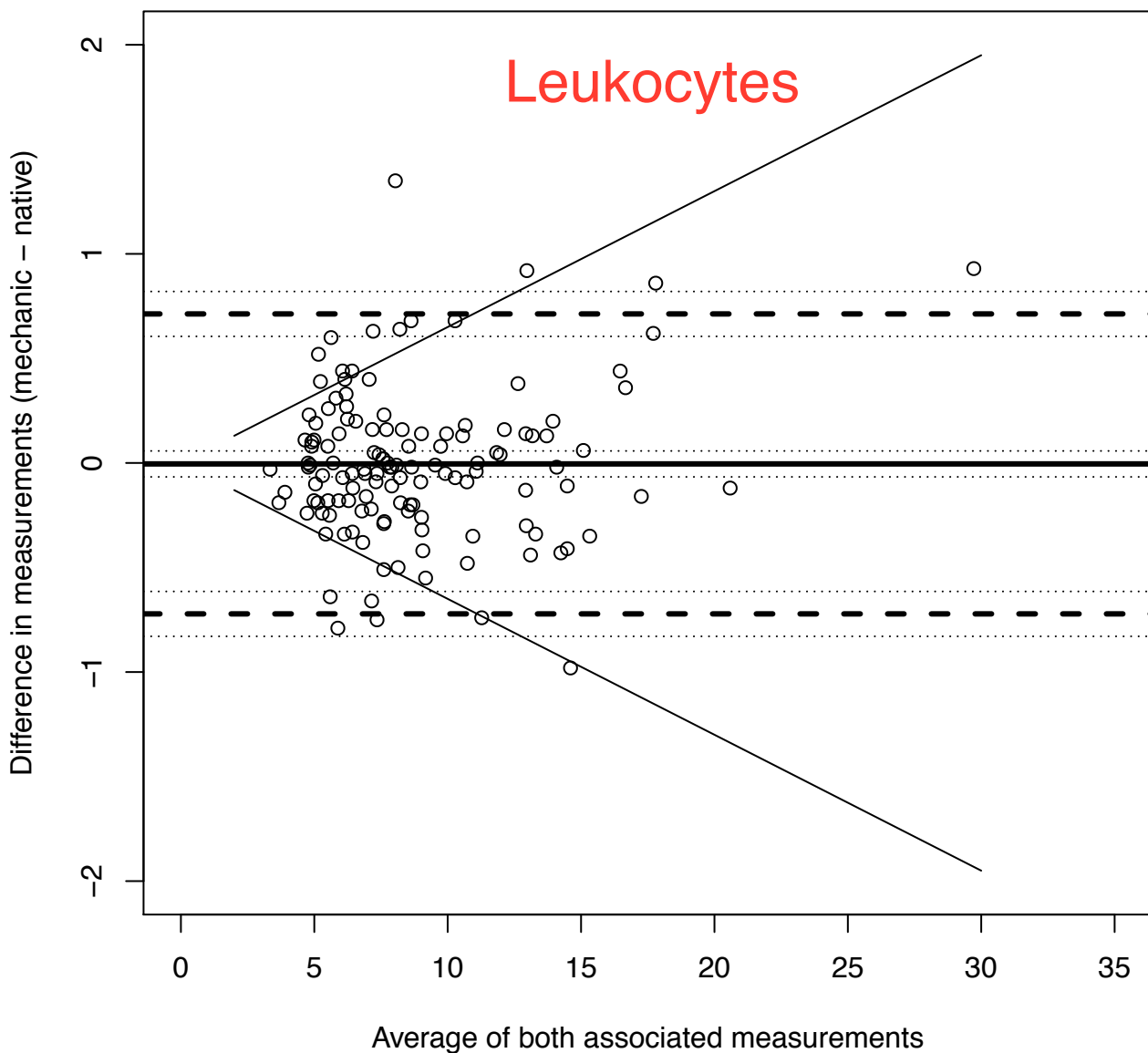

Supplement: Additional file 1: Figure S1. — Leukocytes (Unit: 109/l). Bland-Altman plots for selected biomarkers. The difference in measurements is plotted against the average value of both associated measurements. The margins of accuracy as given by the Rili-BAEK are drawn as sloping lines. Agreement is high. Variations are random and no systemical bias through treatment can be detected. Inaccuracy is well within the limits of the Rili-BAEK and within tolerance of clinical interpretation. (PDF 37 kb) [file 13049_2017_371_MOESM1_ESM.pdf]

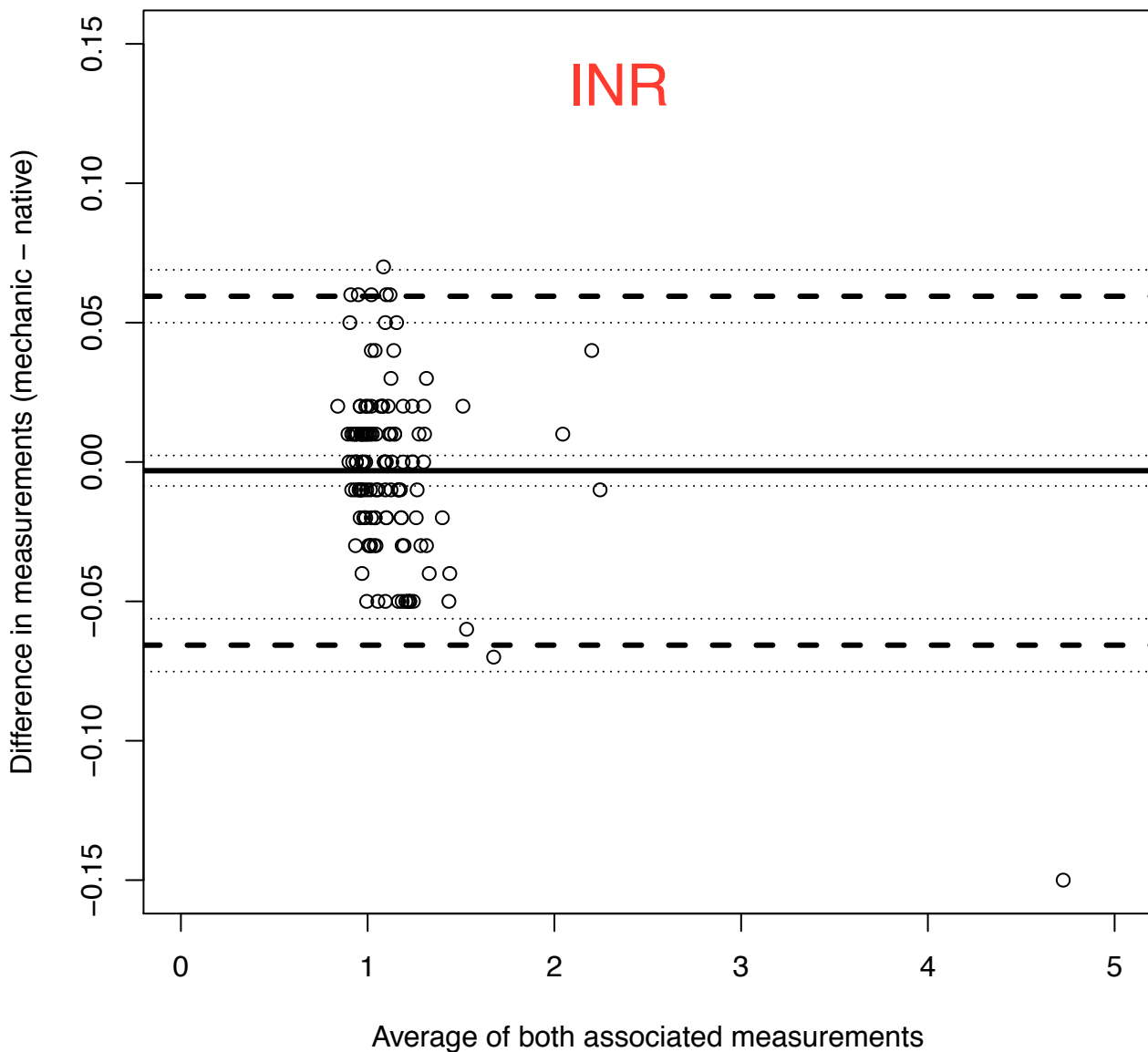

Supplement: Additional file 2: Figure S2. — INR (Unit: not applicable). Bland-Altman plots for selected biomarkers. The difference in measurements is plotted against the average value of both associated measurements. The margins of accuracy as given by the Rili-BAEK are drawn as sloping lines. Agreement is high. Variations are random and no systemical bias through treatment can be detected. Inaccuracy is well within the limits of the Rili-BAEK and within tolerance of clinical interpretation. (PDF 36 kb) [file 13049_2017_371_MOESM2_ESM.pdf]

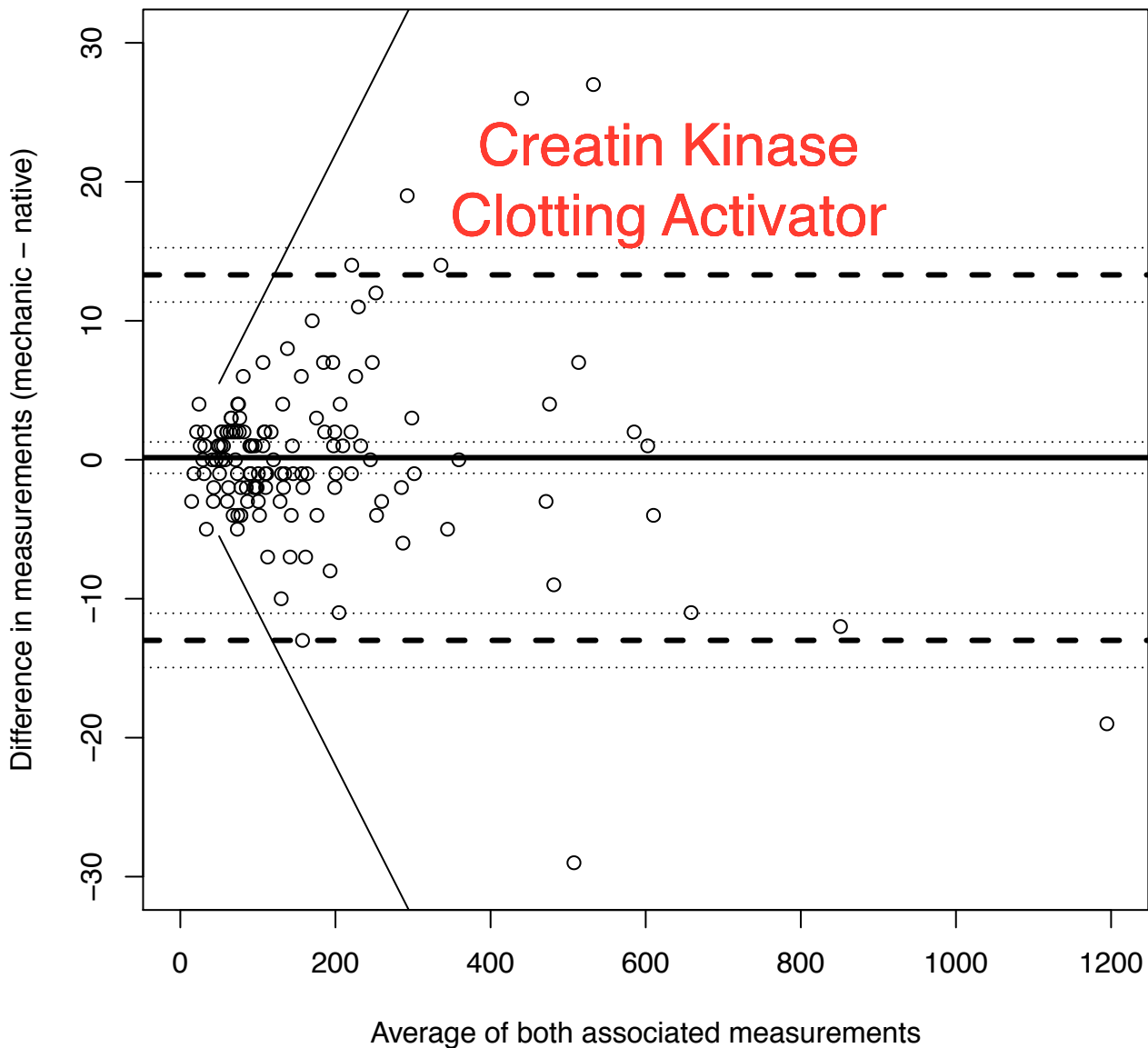

Supplement: Additional file 3: Figure S3. — Creatin Kinase in serum with clotting activator (Unit: U/l). Bland-Altman plots for selected biomarkers. The difference in measurements is plotted against the average value of both associated measurements. The margins of accuracy as given by the Rili-BAEK are drawn as sloping lines. Agreement is high. Variations are random and no systemical bias through treatment can be detected. Inaccuracy is well within the limits of the Rili-BAEK and within tolerance of clinical interpretation. (PDF 63 kb) [file 13049_2017_371_MOESM3_ESM.pdf]

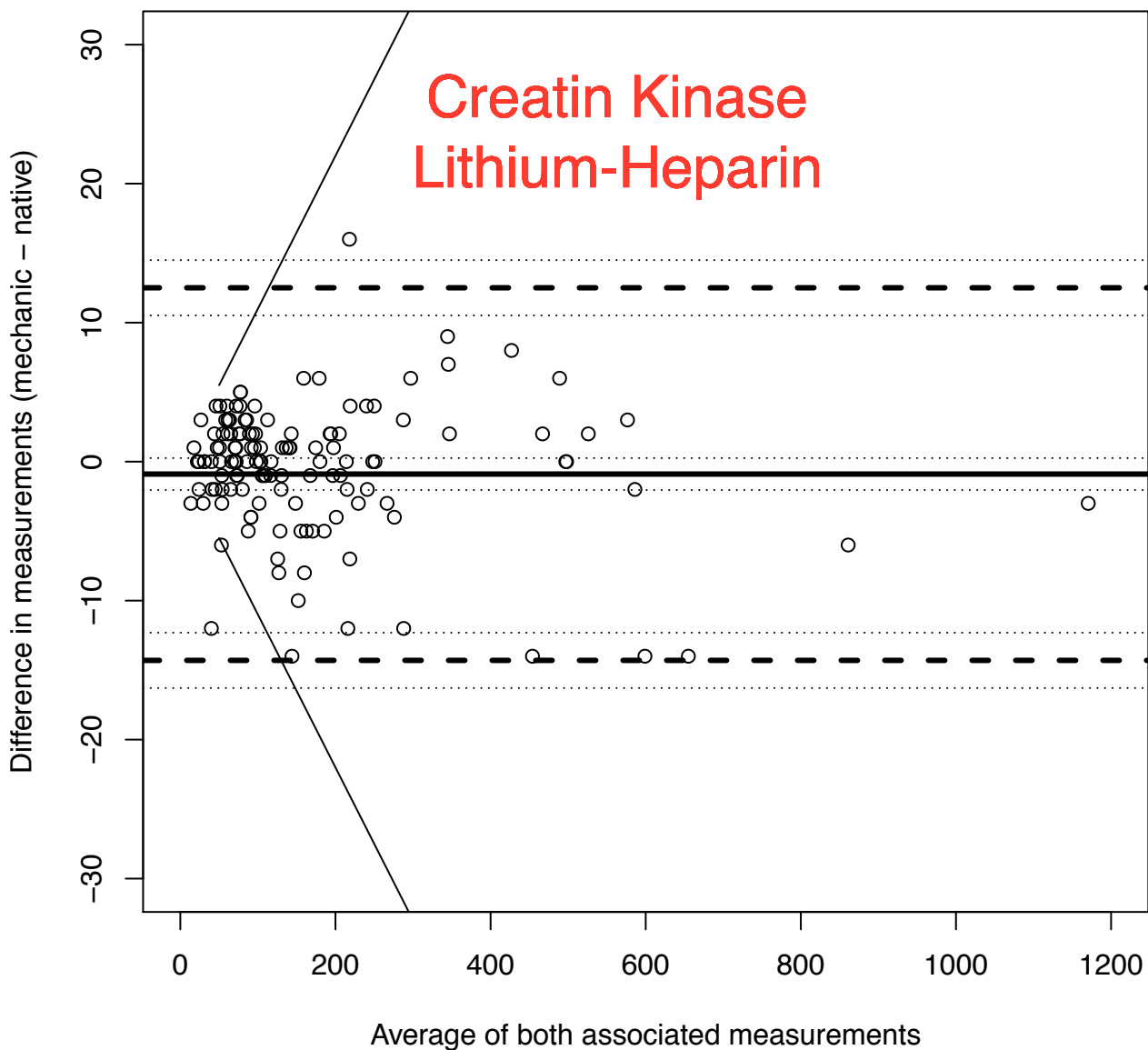

Supplement: Additional file 4: Figure S4. — Creatin Kinase in Lithium-Heparin preserved serum (Unit: U/l). Bland-Altman plots for selected biomarkers. The difference in measurements is plotted against the average value of both associated measurements. The margins of accuracy as given by the Rili-BAEK are drawn as sloping lines. Agreement is high. Variations are random and no systemical bias through treatment can be detected. Inaccuracy is well within the limits of the Rili-BAEK and within tolerance of clinical interpretation. (PDF 94 kb) [file 13049_2017_371_MOESM4_ESM.pdf]

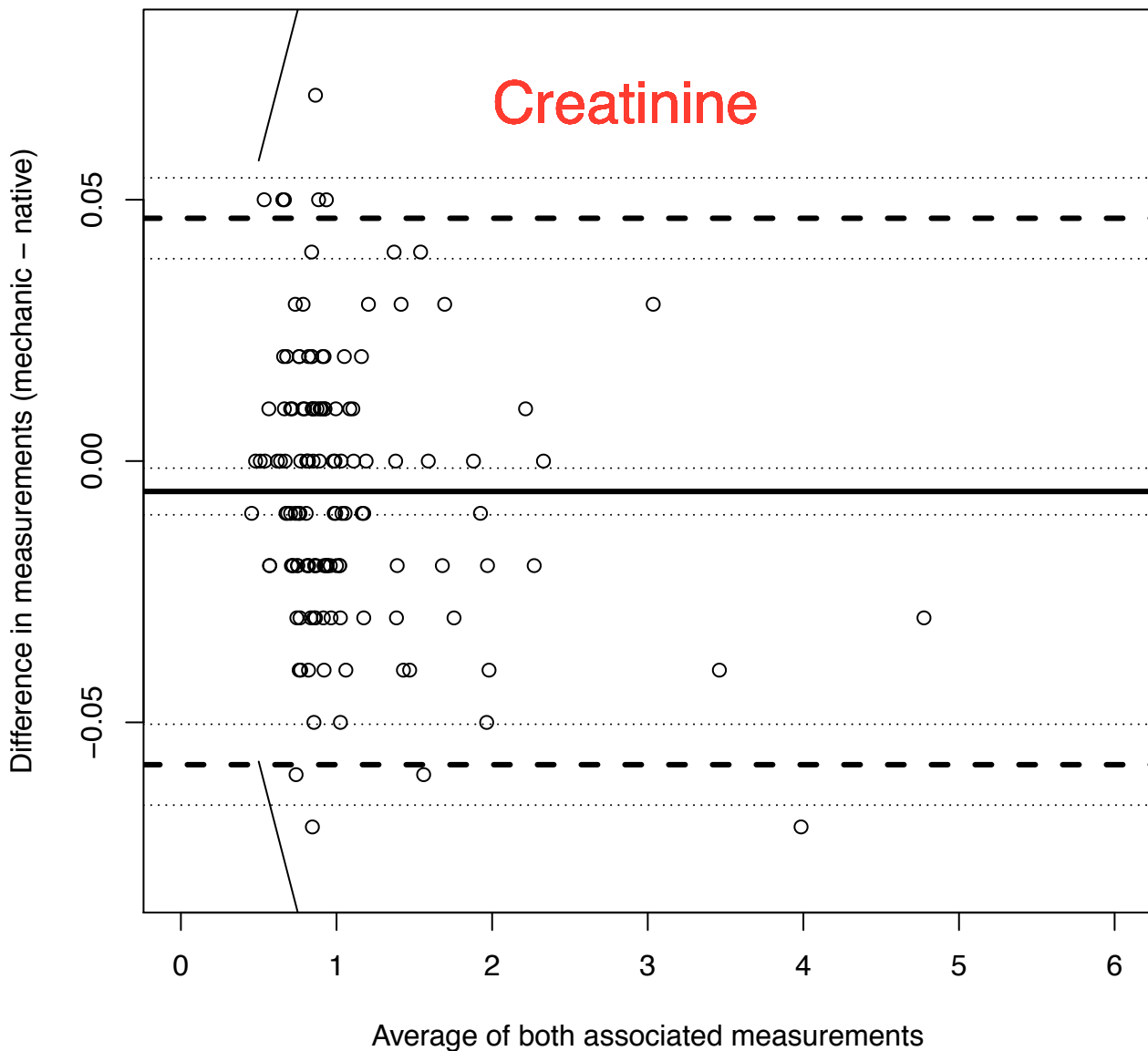

Supplement: Additional file 5: Figure S5. — Creatinine (Unit: mg/dl). Bland-Altman plots for selected biomarkers. The difference in measurements is plotted against the average value of both associated measurements. The margins of accuracy as given by the Rili-BAEK are drawn as sloping lines. Agreement is high. Variations are random and no systemical bias through treatment can be detected. Inaccuracy is well within the limits of the Rili-BAEK and within tolerance of clinical interpretation. (PDF 154 kb) [file 13049_2017_371_MOESM5_ESM.pdf]

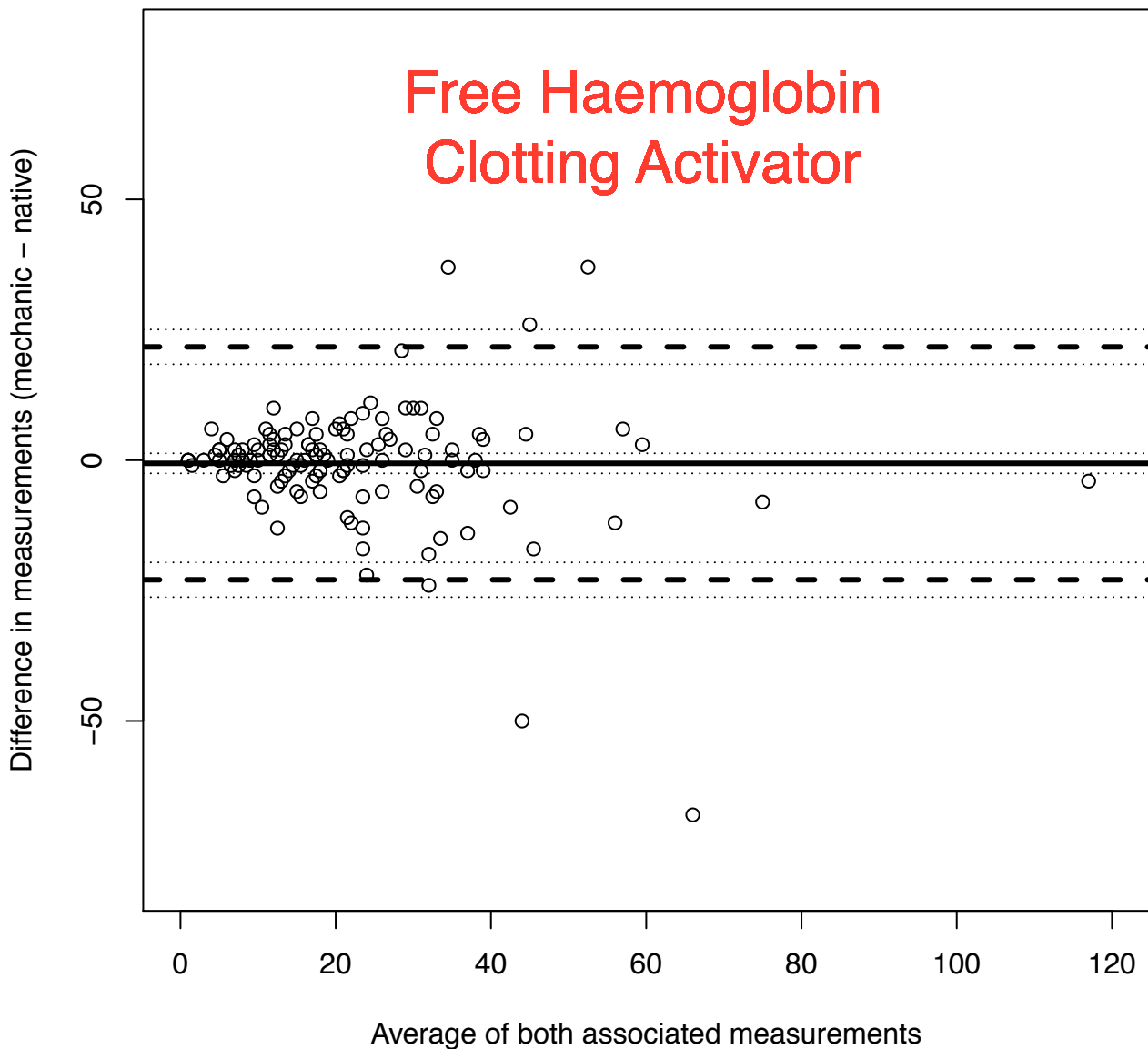

Supplement: Additional file 6: Figure S6. — Free Haemoglobin in serum with clotting activator (Unit: mg/l). Bland-Altman plots for selected biomarkers. The difference in measurements is plotted against the average value of both associated measurements. The margins of accuracy as given by the Rili-BAEK are drawn as sloping lines. Agreement is high. Variations are random and no systemical bias through treatment can be detected. Inaccuracy is well within the limits of the Rili-BAEK and within tolerance of clinical interpretation. (PDF 286 kb) [file 13049_2017_371_MOESM6_ESM.pdf]

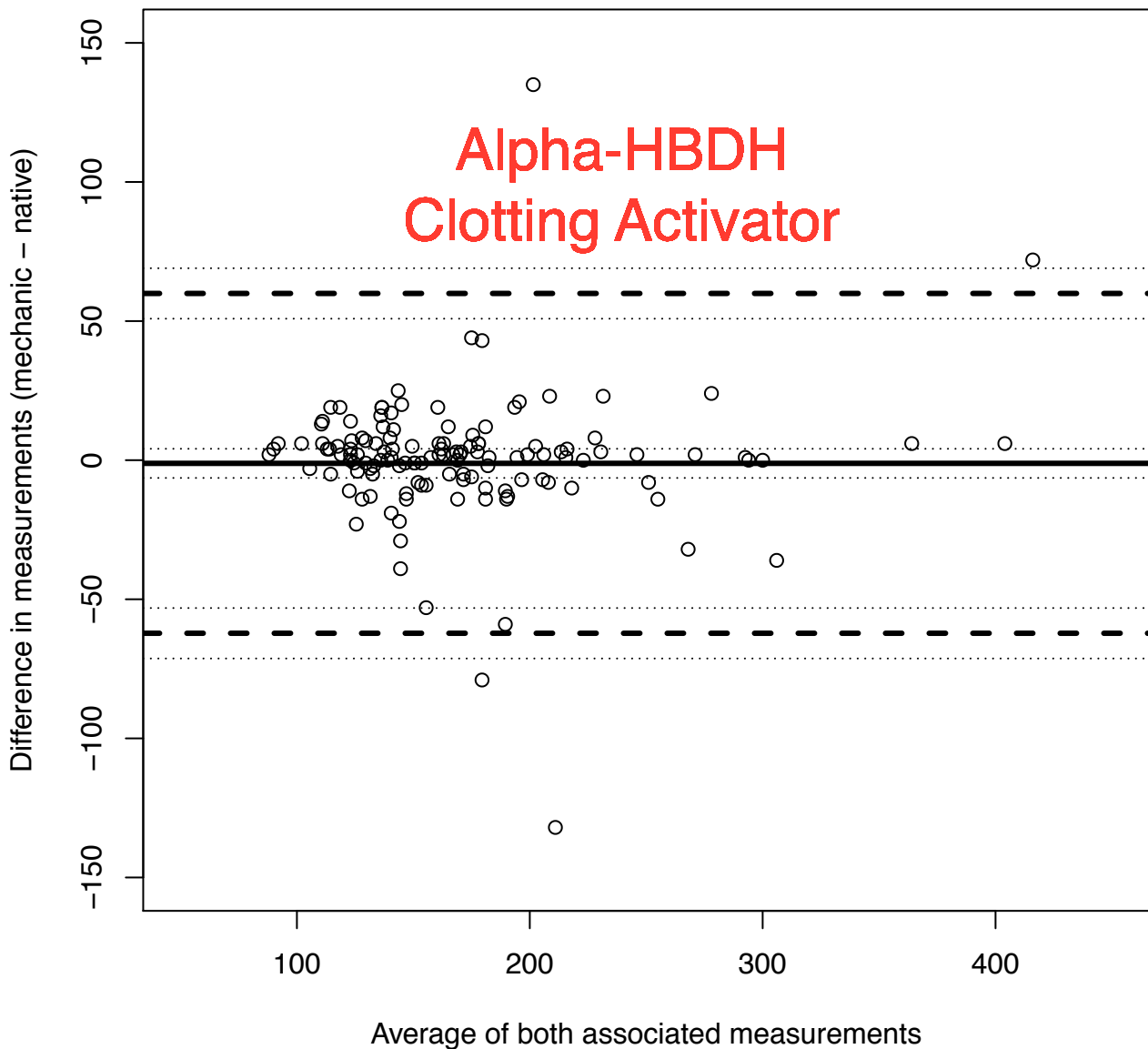

Supplement: Additional file 7: Figure S7. — Alpha-HBDH in serum with clotting activator (Unit: U/l) - Two outlying sample pairs have been excluded from the plot for reasons of better graphical display (No. 1: Native: 1532; Mechanic: 1529; No. 2: Native 357: Mechanic: 135). Bland-Altman plots for selected biomarkers. The difference in measurements is plotted against the average value of both associated measurements. The margins of accuracy as given by the Rili-BAEK are drawn as sloping lines. Agreement is high. Variations are random and no systemical bias through treatment can be detected. Inaccuracy is well within the limits of the Rili-BAEK and within tolerance of clinical interpretation. (PDF 566 kb) [file 13049_2017_371_MOESM7_ESM.pdf]

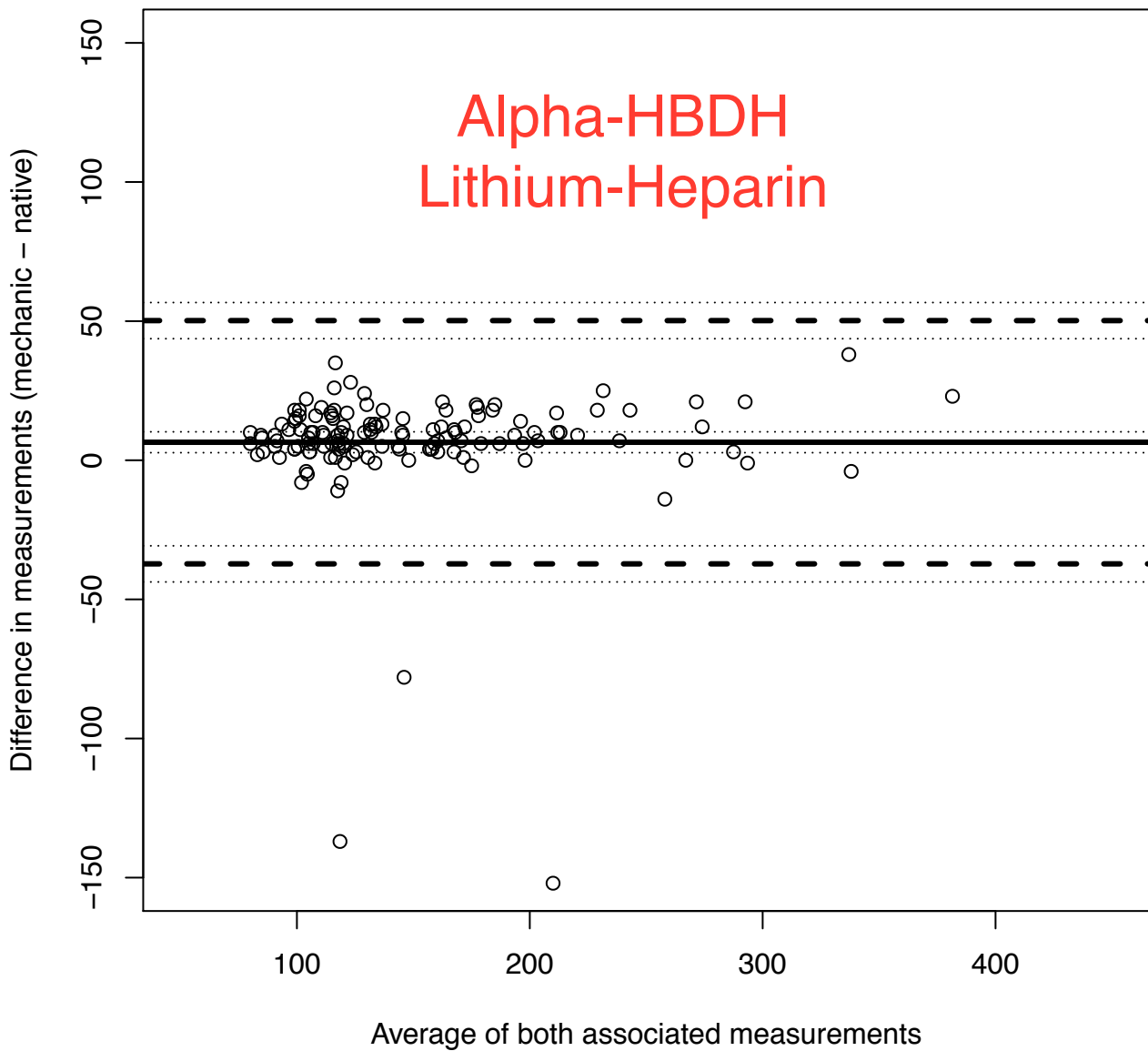

Supplement: Additional file 8: Figure S8. — Alpha-HBDH in Lithium-Heparin preserved serum (Unit: U/l)–One outlying sample pair has been excluded from the plot for reasons of better graphical display (Native: 1526; Mechanic: 1534). Bland-Altman plots for selected biomarkers. The difference in measurements is plotted against the average value of both associated measurements. The margins of accuracy as given by the Rili-BAEK are drawn as sloping lines. Agreement is high. Variations are random and no systemical bias through treatment can be detected. Inaccuracy is well within the limits of the Rili-BAEK and within tolerance of clinical interpretation. (PDF 38 kb) [file 13049_2017_371_MOESM8_ESM.pdf]
